# Predictors of marked weight gain in a population of health care and industrial workers following smoking cessation

**DOI:** 10.1186/s12889-015-1854-7

**Published:** 2015-05-30

**Authors:** Andreas Scherr, Bruno Seifert, Martin Kuster, Anja Meyer, Karl-Olov Fagerstroem, Michael Tamm, Daiana Stolz

**Affiliations:** Clinic of Pneumology and Pulmonary Research, University Hospital Basel, Petersgraben 4, CH-4031 Basel, Switzerland; Industrial Health Service, F. Hoffman- La Roche AG,, Basel, Switzerland; Industrial Health Service, Novartis International AG, Basel, Switzerland; Smokers Information Centre, Fagerstroem Consulting AB, Helsingborg, Sweden

**Keywords:** Weight gain, Post- cessation, Predicting variables, Comprehensive health program

## Abstract

**Background:**

Concerns about postcessational weight gain might hamper rather than encourage smokers to quit smoking.

**Methods:**

We conducted a comprehensive multi-institutional smoking cessation program for health care and industrial workers (n = 654) employed at University Hospital Basel (Switzerland) and two local health industry companies (Novartis International AG, F. Hoffmann-La Roche AG). The program contained counselling with an option of pharmacological support. Changes in body weight were observed during 24 months of follow-up. Factors associated with longitudinal weight gain (>5 % of baseline weight) were identified by cox-regression analysis.

**Results:**

In 51 % of permanent quitters no significant changes of mean body weight were observed after 12 (0.52 kg, SD ±2.87 kg) and 24 months (0.40 kg, SD ± 2.99 kg). Marked weight gain following smoking cessation was characterized by a wide margin of changes. In more than a half of former smokers (58 %) weight increases were moderate (<5 kg), whereas excessive increases (>10 kg) were seen in only 10 % of quitters. Lower baseline BMI (HR 0.60, 95 % CI 0.40- 0.80, p = 0.03), daily consumption of less than ten cigarettes (HR 0.53, 95 % CI 0.27- 0.63, p = 0.04) and ischemic cardiopathy (HR 0.21, 95 % CI 0.07-0.62; p < 0.01) were associated with a lower risk for weight gain. Employees with lower educational levels (HR 2.60, 95 % CI 1.60-5.50, p < 0.01), diabetes mellitus (HR 3.05, 95 % CI 2.20-8.06, p = 0.02) and those smoking to reduce boredom in life (HR 1.68, 95 % CI 1.21-2.33, p < 0.01) were at highest risk.

**Conclusion:**

Marked postcessational weight gain occurs less often than expected, but remains difficult to be predicted. Our findings might be helpful to alleviate weight concerns in the average smoker willing to quit.

## Background

Cigarette smoking is estimated to cause over five millions deaths per year worldwide making it the leading preventable major cause of disease and premature death with a high burden of costs for health care systems [1, 2]. Although smoking cessation is obviously associated with individual health benefits, several barriers addressing physiologic and psychosocial aspects of nicotine addiction often prevent willingness to quit and successful smoking cessation. One of the most important causes of failure in smoking cessation is the fear of excessive weight gain after successful quitting [3, 4]. In comparison with their non-smoking counterparts a lower body weight, a deceleration of age-related weight increase and an 80 percent probability of weight gain occurring within 6 months after quitting is commonly described for smokers [5–8].

Several factors have been examined to identify determinants of postcessational weight gain. These include, although partly controversially discussed, younger age [9], female gender [10], ethnicity, low socioeconomic status [11], baseline weight, determinants of tobacco dependence, a sedentary lifestyle [12] and poor eating habits [13]. In contrast, there is some evidence that both nicotine replacement therapy (NRT) and bupropion alone or prescribed in combination with NRT can at least partly prevent weight gain following smoking cessation [14–16]. Success rates of most smoking cessation interventions remain modest, but may be increased in future through prematurely identifying determinants of weight gain. The main objective of the present study was to explore postcessational weight changes in a first step and thereafter searching for predictors of marked weight gain in an employed population of health care and industrial workers. For this purpose, longitudinal, prospective data for analysis were taken from our comprehensive smoking cessation program [17].

## Methods

### Study design

The study was designed as an investigator- initiated and investigator- driven, open multicentre project which aimed to implementate a comprehensive smoking cessat-ion program in a cohort of employees. It was conducted between May 2005 and January 2009 in Basel, Switzerland. Smoking employees at the University Hospital of Basel (UHBS, Basel, Switzerland) and two local pharmaceuticals companies (Novartis Pharma AG, Basel, F. Hofmann-La Roche AG, Basel) were recruited on a voluntary basis from a total of 17500 employees. All subjects were invited to take part through local advertising such as posters, newsletters and flyers. An additional, standardized and confidential pre-screening questionnaire was sent to all employees of University Hospital Basel. Returned questionnaires were analysed and smokers interested in quitting were contacted by phone through an occupational healthcare advisor. Main criteria of inclusion were willingness to quit smoking, employment at one of the three worksites, a minimum age of 17 years and a daily consumption of at least five cigarettes. All employees provided their written informed consent to a scientific analysis of their data. Prior to the initiation of the program, occupational healthcare advisors of the three study centres received specific training in smoking cessation counselling skills. For this, a standardized manual of smoking cessation training for healthcare professionals was used [18]. Aims, methodology and study design of our smoking cessation intervention are described more in detail elsewhere [17].

The program was designed in accordance with the Swiss and international clinical guidelines for smoking cessation [19, 20]. The local joint ethical committee of the Canton of Basel, Switzerland, approved the project in January 2005 (EKBB 34/05). Participation was on a voluntary basis and written informed consent for scientific evaluation of the program was required from employees prior to enrolment.

### Smoking cessation intervention

The primary intervention consisted of several in site visits within the first 3 months following enrolment. Each visit contained individual face-to-face counselling of at least 15 minutes duration. Counselling support covered standard cognitive- behavioural techniques, such as enhancing motivation, highlighting health benefits, identifying barriers, coping with cravings and relapse prevention. Additionally pharmacological support was provided on a voluntary basis for a total period of 3 months and followed common recommendations of manufacturers and literature regarding dose and delivery systems [21, 22]. Employees were given the choice between either monotherapy or different combinations of nicotine replacement therapy (NRT). Furthermore, bupropion could be added to NRT or was prescribed as monotherapy [17].

After 3 months the primary intervention was completed and all employees had an in-site follow- up at 12 and 24 months, respectively. In addition, telephone contacts asking for smoking status were provided at 6 and 18 months.

Subjects were categorized as abstinent from cigarette smoking by asking self- reported abstinence over the last 30 days. Self-reported abstinence had to be biochemically confirmed by an expired carbon monoxide level of less than 6 ppm measured with a Micro Smokerlyser (Bedfont, Kent, UK). Employees who reported smoking by themselves, had an exhaled carbon monoxide concentration equal or greater than 6 ppb or were lost of follow- up were categorized as relapsed smokers.

### Data collection

Data were collected using a web-based standardized and structured electronic case report form (eCRF) at all three worksites. Clinical relevant variables were assessed at in-site visits. Body weight (in kilograms) was measured for each participant under control of study staff. Employees were weighed with their clothes on, but without wearing shoes, jackets or heavy outer garnets. Height was measured to the nearest 0.5 cm using a tape measure without shoes using a standardized scale. Body weight was followed up by using a standardized spring scale at every in-site visit. The Body mass index (BMI) was calculated as body weight (in Kg) divided by the body height (in m^2^). Weight change was calculated using baseline weight (taken at the individual quit date at visit 3) subtracted from the body weight measured at each consecutive visit.

### Statistical analysis

Baseline characteristics of the whole population and long- term quitters separated in those with and without a marked weight gain following smoking cessation were summarized with means, standard deviations (SD) or proportions. Proportions and frequencies between different groups were compared using the χ_2_- test or the Wilcoxon-rank sum test for not normally distributed variables. Differences in continuous variables were analysed with the two- sample t- test, or with the Mann- Whitney test as appropriate. Marked weight gain was defined as an increase of more than 5 % in reference to the baseline body weight for predictor analysis. Absolute weight chances over times were illustrated in kilograms including means, standard deviations and 95 % confidence intervals. Thereby, quantitative changes of body weight gain were categorized as excessive (more than 10 kg), severe (5–10 kg) and moderate (less than 5 kg) for descriptive statistics (see section results). Longitudinal weight analysis was carried out separately for the whole study population, part- time quitters (abstainers for at least two consecutive visits) and those who remained permanently (over 24 months) abstinent.

In order to detect the effect of nicotine abstinence (versus non- abstinence) on body weight, linear mixed effect models were created. An uni- and multivariable cox- regression analysis to predict the risk of marked weight increase was calculated for several variables of clinical interest. Results are presented as hazard ratios (HR) and 95 % confidence intervals with corresponding p-values. For continuous or ordinal variables hazard ratios had to be based on a meaningful difference of the predicting variable. They were expressed as the ratio from the odds from the 3rd to the 1^st^ quartile representing a typical above average to a typical below average value. All models were adjusted by age, gender and other confounding variables. Due to the exploratory character of the study, no adjustments for multiple comparisons were done. The method for selection of a best subset of variables was based on a stepwise procedure by Akaike Information Criterion (AIC). AIC is a measure of quality of statistical models. Variables were deleted from the whole model until the value of the AIC significantly increased, as previously described [23]. Harrel’s concordance index (C-Index) was calculated with corresponding 95 % confidence intervals to evaluate the concordance between predicted and observed weight gain. All analysis were done using R version 3.0.1. [24].

## Results

### Postcessational weight gain

Assuming a smoking prevalence of about 25 % in the overall swiss population, at least 4250 employees from a total of 17500 employees at the three study centres were estimated to be active smokers [25, 26]. Within our program an estimated 20 % or 885 smoking employees could be recruited initially to quit smoking. 2,555 out of 4449 (57 %) pre-screening questionnaires were returned from employees of University Hospital of Basel. 1,455 (57 %) employees were categorized as non-smokers, 791 employees (31 %) as former smokers and 333 employees (13 %) as active smokers. 253 (76 %) out of 333 active smokers were eligible for enrolment and signed the informed consent. Furthermore, 344 smokers out of 9000 employees and 288 smokers out of 4000 employees entered the program from the worksites F. Hoffman- La Roche AG (Basel, Switzerland) and Novartis Pharma AG (Basel, Switzerland) respectively. Participation rates of recruited smokers were in both centres about 70 %. During the follow-up phase three study subcentres had to be closed due to internal restructuring of the two health companies leading to exclusion of 182 employees. 49 further cases had to be excluded due to missing weight data at several time points in 24, invalid data entries in 3 and missing of relevant covariates in 22 cases. Finally, data from 654 employees were eligible for postcessational weight analysis.

The study population consisted of slightly more male, mid-aged smokers with an average consumption of 11–20 cigarettes day and moderate to severe nicotine dependence on FTND. Descriptive characteristics at baseline for the whole study population and separately illustrated for long-term quitters with or without a 5 % increase of body weight are depicted in Table 1. There were no significant baseline differences of demographic data, prevalence of comorbidities, smoking related variables and different types of pharmacological cessation support with the exception of a slightly lower prevalence of heart disease and higher values of FTND in quitters with marked weight gain. At 24 months of follow- up, 229 employees (35 %) from 654 employees were continuously abstinent. 268 employees (41 %) were categorized as lost-of follow- up. 156 employees (24 %), who were categorized as ongoing smokers or not permanent quitters absolved the whole follow-up of 24 months.

**Table 1:** Baseline characteristics of employees participating in a smoking cessation program

Characteristics	All subjects (*n* = 654)	Long-term quitters (*n* = 229)		
Demographics		Subjects without increase of body weight >5 % (n = 115)	Subjects with Increase of body weight >5 % (n = 114)	*p*-value
Age, years ^a^	42.4 (±9.7)	43.4 (±9.7)	44.2 (±9.31)	0.53
Gender ^b^				0.64
Male	369 (56)	61 (53)	65 (57)	
female	285(44)	54 (47)	49(43)	
Height, cm ^c^	173 (167–180)	173 (168–180)	173 (167–180)	0.76
Weight, kg ^c^	75.6 (64–84)	74.8 (64–83)	74.6 (65–84)	0.45
BMI ^a^	25.1(±4.00)	25.1 (±4.23)	25.4 (±4.44)	0.52
Civil status ^b^				0.47
single	218 (33)	36 (31)	35 (31)	
married	330 (51)	57 (50)	61 (53)	
divorced	95 (14)	19 (16)	18(16)	
widowed	11 (2)	3 (3)	0 (0)	
Education ^b^				0.56
University	141 (22)	35 (31)	28 (25)	
Apprenticeship	466 (72)	73 (64)	77 (68)	
Basic education	41 (6)	6 (5)	8 (7)	
Comorbidities ^b^				
Arterial hypertension	37 (6)	9 (8)	13 (7)	1.00
Coronary heart disease	28 (4)	11 (10)	2 (2)	0.02
Diabetes mellitus	16 (2)	12 (11)	13 (11)	1.00
Malignancy	27 (4)	5 (4)	4 (4)	1.00
Epilepsy	15 (2)	5 (4)	2 (2)	0.45
Psychiatric disorder	91 (14)	24 (21)	13 (11)	0.08
Antidepressant medication	112 (17)	23 (20)	16 (14)	0.31
Satisfaction with Life Scale (Qol), points ^c^	26 (24–30)	27 (25–31)	26 (23–30)	0.82
Respiratory symptoms ^b^				
Cough	253 (39)	34 (30)	38 (34)	0.61
Sputum	176 (27)	25 (22)	26 (23)	0.97
Wheezing	124 (19)	26 (23)	17 (15)	0.19
Breathlessness at rest	44 (7)	12 (10)	10 (9)	0.84
Breathlessness on exertion	288 (44)	47 (41)	45 (39)	0.94
Smoking-related history				
Age at starting smoking, years ^a^	17 (±3.7)	17 (±3.8)	17 (±3.5)	1.00
Prior attempts to quit ^a^	3.0 (±3.5)	2.9 (±3.3)	3.1 (±3.8)	0.78
Duration longest nicotine abstinence, months ^a^	20 (±51)	21 (±45)	20 (±54)	0.69
Years, smoked ^a^	22 (±8.5)	21 (±10.3)	22 (±8.5)	0.85
Number of cigarettes/day ^b^				0.29
0-10	82 (13)	22 (19)	12 (11)	
11-20	329 (50)	59 (51)	67 (59)	
21-30	184 (28)	28 (24)	27 (24)	
>30	58 (9)	6 (5)	8 (7)	
FTND, points ^a^	4.5 (±2.40)	3.7 (±2.40)	4.5 (±2.30)	0.02
Intensity of craving, (VAS 0–100) ^a^	57 (±32)	58 (±32)	55 (±21)	0.09
Exhaled CO, ppm ^a^	22.6 (±14.7)	20.2 (±15.1)	22.0 (±13.9)	0.38
Pharmacological support				0.91
NRT				
Mono	82 (13)	12 (10)	11 (10)	
Combined	124 (19)	24 (22)	21 (19)	
Mono + Bupropion	92 (14)	12 (10)	13 (12)	
Combined + Bupropion	265 (42)	52 (45)	57 (50)	
Bupropion	42 (6)	5 (4)	5 (4)	
Counselling only	47 (6)	10 (9)	6 (5)	

Data are illustrated for all subjects and permanent abstainers with and without significant change in body weight gain (def. Increase of body weight > 5 % as compared to baseline within 2-years)

^a^ Values are means ± SD, ^b^ absolute numbers (%) or ^c^ medians with interquartile ranges, BMI = Body mass index; FTND = Fagerstroem test for nicotine dependence, exhaled CO = exhaled carbon monoxide; NRT = Nicotine replacement therapy

**Fig. 1:**
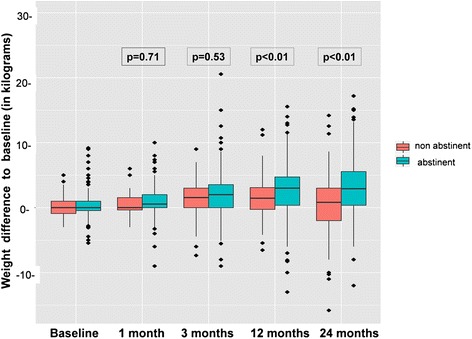
Longitudinal effect of nicotine abstinence on weight changes as compared to baseline. Postcessational weight gain of quitters was significant increased after 12 months as compared to relapsed smokers (n = 654)

Mean weight gain over time in the whole study population (n = 654) was 0.80 kg (±1.20 kg) at 1 month, 1.84 kg (±1.50 kg) at 3 months, 2.04 kg (±1.90 kg) at 12 months and 1.98 kg (±2.20 kg) after 24 months, respectively. At the time of 12 and 24 months, weight changes from permanent abstainers were significantly different from baseline as compared with relapsed smokers (p < 0.01). Weight differences over time splitted for abstinence vs. non- abstinence are illustrated in Fig. 1.

In about a half of permanent quitters (n = 114, 49 %) marked weight gain occurred. Average longitudinal increase of body weight in these 114 subjects was 1.30 kg (SD ±2.40 kg) at 1 month, 2.90 kg (SD ±2.91 kg) at 3 months, 5.07 kg (SD ±3.50 kg) after 1 year and remained stable thereafter with 5.15 kg (±4.14 kg) at 2 year follow-up (p < 0.01). Excessive weight gain (>10 kg) occurred in 11 (10 %), severe (5–10 kg) in 36 (32 %) and moderate gain (less than 5 kg) in 67 (58 %) out of 114 permanent quitters. By comparison, in 51 % of long-term quitters (n = 115) no significant changes of mean body weight were observed [0.07 kg (±1.69 kg) at 1 month, 0.68 (±2.23 kg) at 3 months, 0.52 kg (±2.87 kg) at 12 months and 0.40 kg (±2.99 kg)] after 24 months. The risk of smoking relapse was significantly lower for smokers with an weight gain between 0 and 10 kg as compared to the subgroup with excessive weight gain (>15 kg) ( HR 0.32; 95 % CI 0.25-0.42, p < 0.01).

### Predictors of postcessational weight gain

Univariate- and multivariate Cox regression analysis assessing characteristics potentially associated with marked weight gain (defined as 5 % increase of baseline weight) after smoking cessation are summarized in Table 2. Taken demo-graphic characteristics into account, only a lower baseline weight (BMI 20 vs. 30) was associated with a lower chance of postcessational weight gain (HR 0.60, 95 % CI 0.40-0.80, p = 0.03). In contrast to univariate analysis (HR 1.42, 95 % CI 1.07-1.87, p = 0.01), female gender was not an independent predictor for marked weight gain on multivariate analysis (HR 1.40, 95 % CI 0.93-2.10, p = 0.11). Similarly, age of employees (30 vs. 50 years) was also not predictive for weight gain (HR 1.00, 95 % CI 0.97-1.03, p = 0.94). In contrast, a lower level of education, namely basic education or apprenticeship, were strongly associated with an increase of body weight (basic education HR 2.90 95 % CI 1.39-4.83, p < 0.01, apprenticeship HR 2.60, 95 % CI 1.60-5.50, p < 0.01). From present comorbidities a concomitant diagnosis of diabetes mellitus or ischemic cardiopathy was found to be independently associated with weight changes. Thereby, diabetes mellitus was a strong risk factor (HR 3.05, 95 % CI 2.20-8.06, p = 0.02), whereas employees with coronary disease seemed to have a lower risk for weight gain (HR 0.21, 95 % 0.07-0.62, p < 0.01). Smoking related variables of nicotine dependency, namely FTND or Craving scores, revealed no differences in weight changes, although at least a daily consumption of less than ten cigarettes per day appeared to be protective for excessive weight gain (HR 0.53, 95 % CI 0.27-0.63, p = 0.04). The association of cigarette smoking to reduce feelings of boredom resulted in significant higher body weight after quitting (HR 1.68, 95 % CI 1.21-2.33, p < 0.01).

**Table 2:** Univariate and multivariate cox- regression analysis to predict postcessational weight gain of more than 5 % of baseline body weight within 24 months (n = 654)

*n* = 654		Univariate cox -regression analysis			Multivariate cox-regression analysis	
Variables	HR	95 % CI	*p*	HR	95 % CI	*p*
Demographics						
Age (30 vs. 50 years)	1.00	0.99-1.02	0.59	1.00	0.97-1.03	0.94
BMI (20 vs. 30)	0.45	0.35-0.85	**0.02**	0.60	0.40-0.80	**0.03**
Gender						
Male	----	----	----	---	---	---
Female	1.42	1.07-1.87	**0.01**	1.40	0.93-2.10	0.11
Civil status						
Single)	----	----	----	---	---	---
Married	0.97	0.64-1.46	0.88	---	---	---
Divorced	0.78	0.44-1.39	0.46	---	---	---
Widowed	1.40	0.46-4.17	0.55	---	---	---
Education						
University	----	----	----	---	---	---
Apprenticeship	1.97	1.20-3.24	**<0.01**	2.60	1.60-5.50	**<0.01**
Basic education	3.02	1.60-5.36	**<0.01**	2.90	1.39-4.83	**<0.01**
Comorbidities						
Arterial Hypertension	0.99	0.52-1.91	1.00	1.05	0.51-2.18	0.89
Coronary heart disease	0.34	0.12-0.95	**0.02**	0.21	0.07-0.62	**<0.01**
Diabetes mellitus	2.22	1.11-6.01	**0.04**	3.05	2.20-8.06	**0.02**
Malignancy	1.01	0.44-2.32	0.98	0.61	0.25-1.50	0.28
Epilepsy	1.08	0.78-1.86	0.78	1.42	0.5-1.32	0.45
Antidepressant medication	0.93	0.62-1.40	0.73	0.89	0.54-1.46	0.64
Psychiatric disorder	1.19	0.78-1.80	0.41	1.07	0.62-1.85	0.79
Satisfaction with Life (Qol), (points)	0.98	0.95-1.01	0.26	0.98	0.95-1.02	0.42
Respiratory Symptoms						
Cough	1.02	0.99-1.04	0.18	1.00	0.90-1.08	0.42
Sputum	1.50	1.08-2.07	**0.01**	1.32	0.89-1.95	0.17
Wheezing	1.23	1.10-2.34	0.26	1.31	1.09-1.17	0.35
Breathlessness at rest	1.59	0.94-2.69	0.09	1.29	0.65-2.55	0.47
Breathlessness at exertion	1.04	0.78-1.37	0.80	0.93	0.66-1.30	0.66
Smoking related history						
Number of cigarettes/day						
0-10	0.56	0.34-0.93	**0.02**	0.53	0.27-0.63	**0.04**
21-30	0.90	0.64-1.27	0.55	0.74	0.48-1.14	0.18
>30	1.34	0.81-2.20	0.25	0.97	0.48-1.96	0.94
FTND (6 vs. 3 points)	1.09	1.03-1.16	**0.01**	1.09	0.98-1.21	0.12
Intensity of craving (VAS 100 vs. 50)	1.00	0.99-1.01	0.35	1.00	0.99-1.00	0.50
Exhaled CO, ppb	1.07	0.99-1.02	0.11	0.99	0.99-1.01	0.84
Motivation to quit (VAS 100 vs.50)	1.00	0.99-1.01	0.48	1.00	0.99-1.02	0.63
Motivation for smoking						
Weight control	1.10	0.79-1.55	0.57	1.02	0.65-1.59	0.94
Dependency	0.91	0.60-1.38	0.66	0.91	0.61-1.38	0.66
Mood control	1.32	0.93-1.86	0.12	0.97	0.63-1.48	0.87
Boredom	1.50	1.13-1.99	**<0.01**	1.68	1.21-2.33	**<0.01**
Unwillingness to quit	1.40	1.02-1.92	**0.04**	1.25	0.84-1.84	0.27
Relaxation	1.02	0.76-1.36	0.92	0.90	0.62-1.31	0.60
Obstipation	1.12	0.70-1.78	0.64	0.92	0.49-1.71	0.79
Pharmacological support						
Counselling only	----	----	----		---	---
NRT						
Mono	0.82	0.41-1.68	0.59	---	---	---
Combined	1.09	0.57-2.1	0.80	---	---	---
Mono + bupropion	0.99	0.50-1.97	0.98	---	---	---
Combined + bupropion	1.75	0.95-3.19	0.07	---	---	---
Bupropion	1.43	0.68-3.02	0.34	---	---	---

The reference category for discontinuous or ordinal variables is given in parentheses. For continuous or ordinal variables, OR had to be based on meaningful differences of the predicting variable. BMI = Body mass index; FTND = Fagerstroem test for nicotine dependence; exhaled CO = exhaled carbon monoxide; NRT = Nicotine replacement therapy

**Fig. 2:**
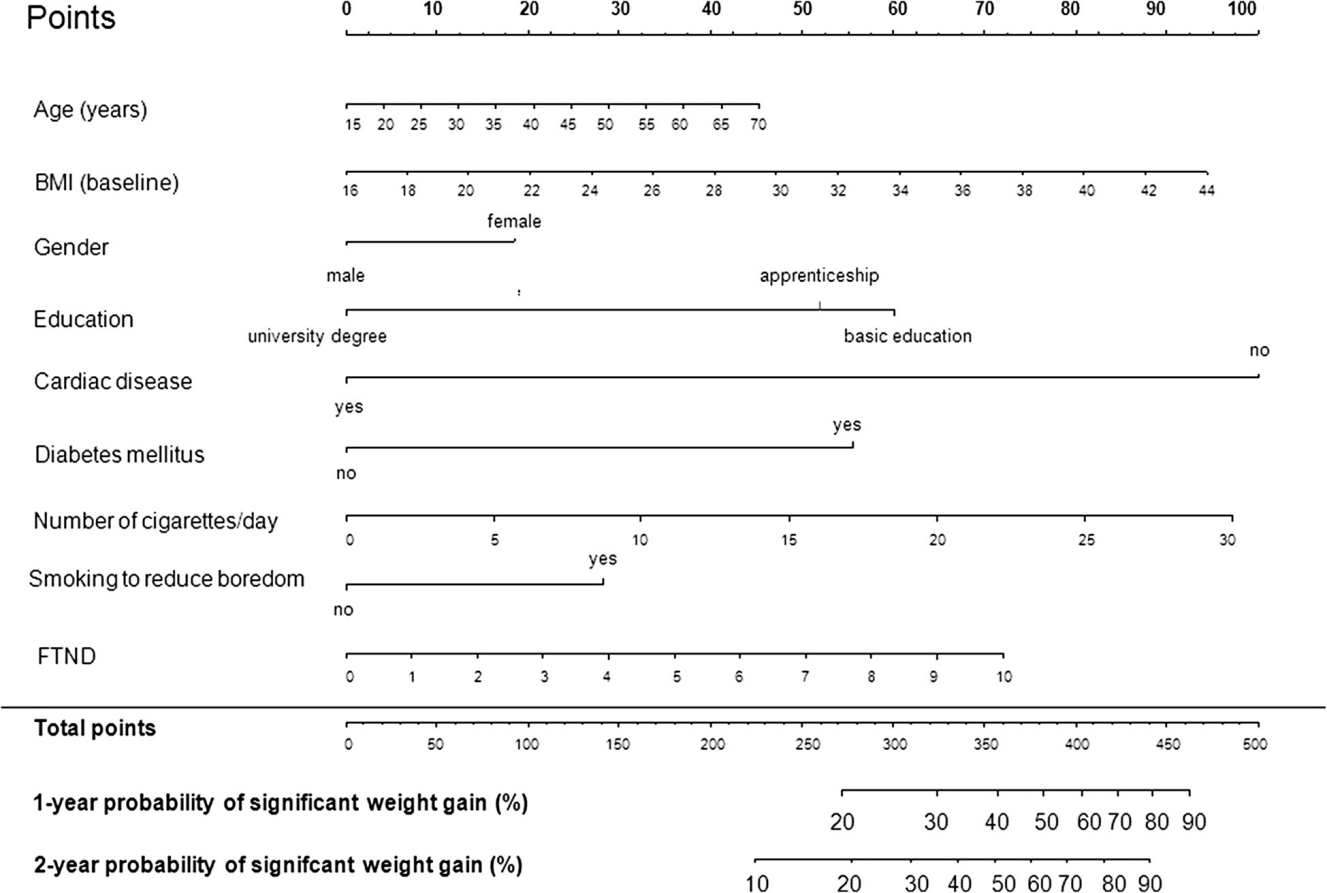
“Best subset regression” model for weight gain predicting variables. To use the nomogram, locate the first variable. Draw line straight up to the points axis to determine the number of points received for the variable. This has to be repeated for all other variables and points achieved for each variable have to be summarized. The sum of these numbers is located on the total point axis and a line drawn has to be drawn down to determine the likehood of 1- and 2- probability of postcessational weight gain. BMI = Body Mass Index, FTND = Fagerstroem Test for Nicotine Dependence

Differences in weight gain could not be observed when different types of nicotine replacement therapy with or without bupropion were assessed individually in comparison with the counselling only option. A total of 9 variables were selected for a best subset regression model and a nomogram to estimate one and two year probability for marked weight gain was constructed (c- index. 0.68, 95 % CI [0.63; 0.72]) (see Fig. 2).

## Discussion

To our knowledge, the present study is one of the first of its kind to examine long-term weight gain in order to identify predicting factors in a cohort of healthy mid-aged workers participating in a comprehensive worksite related health intervention.

As a main finding of the present study, we observed no significant weight changes following smoking cessation in more than a half of long-term quitters. In the subgroup of long-term with marked weight gain we revealed a mean weight increase of 5 kg after quitting with a high variability between individuals. We were also able to identify some determinants which are potentially useful to prematurely identify smokers at greatest risk.

The relationship between smoking cessation and consecutive weight gain is considered to be complex in nature and most likely multifactorially conditioned. In line with previous research, smoking cessation was clearly associated with substantial weight gain in many smokers of our population. Thereby it should be mentioned that the frequency, the amount and pattern of weight gain differed widely between investigated populations in the past [27, 28]. In general, assessment of postcessational weight changes is made complex by several facts. The majority of healthy adults in developed countries tend to gain weight with age as it has be shown in several large population studies [10, 29]. More recent data have also confirmed this observation in a smoking population [30]. Furthermore, the individual body weight might also be relevant influenced by different cultural and nutritionals factors [31, 32]. In addition, studies in homozygotic twins have demonstrated that the postcessational weight gain might be in large a part of compensation for underweight in the smoking twin compared to the non-smoking twin [33]. Therefore, a genetic component of individual body weight might also affect postcessational weight gain.

Finally, contrary reports about weight gain following smoking cessation could also be explained –at least to some extent- by several methodological reasons including the use of only self- reported height and weight, failure to biochemically confirm smoking status, missing control- groups, using more often point- versus continuous abstinence and limited follow-up periods [34].

Relevant weight gain might both negatively affect the outcome of a quit attempt and at least party attenuate the health benefits of smoking cessation due to an increased probability for metabolic and cardio-vascular disorders in high-risk subjects [35, 36].

We found a mean weight gain of 5 kg occurring in the first year after quitting, which is in line with various earlier reports describing an increase between 4 to 6 kilograms occurring within the first year in continuously abstinent outpatient smokers [4, 37–40]. As one might expect, we observed greater weight gain over time in permanent abstainers as compared to non-sustained quitters and ongoing smokers. Significant gain of body weight occurred mostly within the first year post quitting and showed thereafter a tendency to stabilize until 24 months. Although evidence whether weight increase beyond one year continues is conflicting, our observation stands in agreement with some newer investigations [41–43].

Some randomized clinical studies postulated that both NRT and bupropion might prevent or at least attenuate weight gain [39, 44, 45]. However, other investigations could not confirm this finding [40, 46]. In the present study, we found no differences in weight changes post- quitting in regards to several modalities of nicotine replacement with or without bupropion as compared to counselling advice only. Nevertheless, it should be mentioned, that our finding was made in a non- randomized, “real-life” setting with unmatched treatment groups. The non-effect of NRT on weight might be therefore due to a self-selection of NRT by heavier smokers with an overall higher risk for weight gain. Furthermore, we did not provide pharmacological support with varenicline, which might be more effective in attenuating weight gain than NRT based on latest findings [47].

Postcessational weight gain is considered both to negatively affect the outcome of a quit attempt and at least party attenuate the health benefits of smoking cessation due to an increased probability for metabolic and cardio-vascular disorders in high- risk subjects [35, 36]. Data from the present study showed that smokers with excessive postcessational weight gain (>10 kg) had a significant higher rate of smoking relapse. Therefore, meaningful predictor variables would be helpful to identify smokers at risk for postcessational weight gain. Predictors identified by existing reports have a number of limitations including restricted generality (data were often collected in clinical populations), which probably differ in weight changes as compared to outpatient, “healthy” smokers [48–51]. In addition, results of population-based investigations often relied on retrospective reports without any objective assessment of weight and smoking status. Additionally, long-term success rates of cessation interventions remained often low. In the present study weight increases revealed a broad range of variation with moderate weight gain (<5 kg) in 58 % and excessive weight gain (>10 kg) in only 10 % of former smokers respectively [11, 37]. Therefore many tested predictor variables for a 5 % increase of baseline weight might be therefore inaccurate in the individual case.

A lower educational level was the strongest risk factors for weight gain after quitting in the present study. Although data describing the influence of socioeconomic variables on body weight gain are scarce, adopted health habits including limited resources for changing nutritional intake and a more sedentary lifestyle in less educated smokers may contribute to relevant weight gain following smoking cessation [11]. Addressing motivational factors, employees who reported to smoke for reducing boredom in their life were significant more vulnerable for weight increase. This may represent an increased caloric intake as vicarious satisfaction. In contrast, employees who reported to utilize smoking as weight control practice had surprisingly neither an increased risk to gain weight after smoking cessation nor lower abstinence rates, although this subgroup of smokers was associated with higher nicotine dependence and lower resources of self- efficacy to control body weight [52]. This observation can’t be fully explained, because data about important confounding variables like restraint eating or increased physical activities during quit attempts were not collected in the present study. Furthermore, the association between individual weight concerns and weight gain after cessation remains controversially discussed in literature [53, 54].

The subgroup of subjects suffering from diabetes had a greater change for weight gain. This seems to be particularly relevant due to the potentially elevated risk for uncontrolled metabolic situation, morbidity and mortality following smoking cessation [55, 56].

The lower risk of weight gain in subjects with coronary heart disease seems surprising at first glance. We assume that employable subjects with coronary heart disease might display a higher alertness for healthier lifestyles. We didn’t found any related articles in order to scientifically substantiate this observation. Additionally, the sample size participants with coronary heart disease (n = 28) was quite low, so the strength of this observation needs further proof due to a low statistical power.

In accordance with previous investigations, smokers with a lower amount of cigarettes smoked per day at baseline seem to be less vulnerable for weight gain suggesting that lower levels of nicotine dependence may be protective against body weight increase [40, 57]. Importantly, it should be mentioned, that our population consisted mainly of low and medium dependent smokers (FTND mean: 4.42 pts, SD ±2.30) and therefore other determinants of nicotine dependence, namely FTND, carbon monoxide and intensity of craving might have failed to independently predict weight gain. The observation, that individual characteristics like age and gender showed no association with postcessational weight gain are in line with some, but not all earlier reports [11, 12, 58, 59]. Our findings are also in accordance with some earlier investigations showing that smokers with normal baseline BMI (20 kg/m^2^) are less vulnerable for marked gain than those with higher baseline weight (BMI 30 kg/m^2^) [58, 60].

The present study has some strengths and limitations. The strengths consist of its community based approach and a high rate of successful long-term quitters (over 35 % at 24 months). In line with a more recent published study we also analysed weight changes for continuous abstainers, part-time quitters and ongoing smokers at different points of time through a longitudinal model [40]. The later aspect ensured that weight changes of all participants were taken into account and not only weight changes from those who remained permanently abstinent or completed the whole follow-up.

One of our most important limitations is the absence of a control group of lifelong non- smokers that would certainly have added to the strength of the study. Nevertheless, in contrast to many similar studies a relevant number of 156 unsuccessful smokers was followed up for 24 months. These participants represented a kind of internal control group enabling us to better estimate the impact of permanent smoking cessation on long-term body weight.

In summary marked weight gain following smoking cessation occurred in less than a half of smokers following smoking cessation. The majority of smokers intending to quit smoking has concerns about postcessational weight gain and the experience of a minority of quitters who gain large amounts of weight may discourage them from quitting. Therefore, it seems important that health care providers alleviate weight concerns and encourage smokers for a quit attempt. Numerous demographic, socioeconomic, smoking related, cultural and even genetic factors were associated with postcessational weight gain. Therefore, it seems improbable that a single or even a subset of variables as demonstrated by the moderate performance of our multidimensional model can predict individual weight gain following smoking cessation. Nevertheless, early recognition of those smokers at risk for excessive weight gain could enhance the success rate of smoking cessation interventions. Further longitudinal well- designed studies are needed to further characterize the determinants of smokers who are particularly vulnerable for weight gain.

## Conclusion

The present study is one of the first of its kind investigating postcessational weight changes in a large community of health care and industrial workers participating on a comprehensive smoking cessation intervention. One of the main findings of our investigation is the observation that marked weight gain following smoking cessation occurs less often than expected. Consistent with previous reports, long- term abstinence was associated with a mean weight gain of 5 kilograms, which occurred in continuous quitters within 1 year. Although some determinants of weight gain could be identified, accurate prediction using a clinical set of characteristics remains difficult in the individual case.

## Abbreviations

CO: Carbon monoxide
COPD: Chronic obstructive pulmonary disease
eCRF: Electronic case report form
FTND: Fagerstroem test for nicotine dependence
VAS: Visual analogue scale
NRT: Nicotine replacement therapy
HR: Hazard ratio
95 % CI: 95 % confidence interval
IQR: Interquartile range
SD: Standard deviation
